# Invasive therapy versus conservative therapy for patients with stable coronary artery disease: An updated meta‐analysis

**DOI:** 10.1002/clc.23592

**Published:** 2021-03-20

**Authors:** Aviral Vij, Kameel Kassab, Hitesh Chawla, Amandeep Kaur, Vamsi Kodumuri, Neeraj Jolly, Rami Doukky

**Affiliations:** ^1^ Division of Cardiology Cook County Health Chicago Illinois USA; ^2^ Department of Medicine Rush Medical college Chicago Illinois USA; ^3^ Division of Cardiology MedStar Union Memorial Hospital Baltimore Maryland USA; ^4^ Department of Pathology University of Chicago‐Northshore Evanston Illinois USA; ^5^ Division of Cardiology Ascension All Saints Hospital Racine Wisconsin USA; ^6^ Division of Cardiology Rush University Medical Center Chicago Illinois USA

**Keywords:** coronary artery bypass grafting, medical therapy, percutaneous coronary intervention, stable coronary artery disease

## Abstract

**Background:**

Heart disease remains the leading cause of death in the United States. Although there are clear indications for revascularization in patients with acute coronary syndromes, there is debate regarding the benefits of revascularization in stable ischemic heart disease. We sought to perform a comprehensive meta‐analysis to assess the role of revascularization compared to conservative medical therapy alone in patients with stable ischemic heart disease.

**Hypothesis:**

There is no significant difference in all‐cause mortality or cardiovascular mortality between invasive and medical arms.

**Methods:**

We performed a systematic literature search from January 2000 to June 2020. Our literature search yielded seven randomized controlled trials. We analyzed a total of 12 013 patients (6109 in revascularization arm and 5904 in conservative medical therapy arm). Primary outcome was all‐cause mortality. Secondary outcomes included major adverse cardiac events (MACE) (death, myocardial infarction [MI], or stroke), cardiovascular mortality, MI, and stroke. Additional subgroup analysis for all‐cause mortality was performed comparing percutaneous coronary intervention (PCI) with bare metal stent versus conservative therapy; and PCI with drug eluting stent versus conservative therapy.

**Results:**

There was no statistically significant difference in primary outcome of all‐cause mortality between either arm (odds ratio [OR] = 0.95; 95% CI [confidence interval], 0.83 to 1.08; *p =* .84). There were statistically significant lower rates of MACE (death, MI or stroke) in the revascularization arm when compared to conservative arm.

**Conclusions:**

Our analysis did not show any survival advantage of an initial invasive strategy over conservative medical therapy in patients with stable coronary artery disease (CAD).

## 1 INTRODUCTION

Benefits of revascularization with percutaneous coronary intervention (PCI) or coronary artery bypass graft surgery (CABG) for patients presenting with acute coronary syndrome (ACS) have been clearly established. Multiple trials^1^, ^2^ have shown improved overall survival and reduction in recurrent myocardial infarction (MI) in patients with ACS. PCI leads to effective restoration of vessel patency, reduced re‐occlusion, and improved residual left ventricular function. This evidence culminated in revascularization for ACS becoming a class I indication as per 2011 ACCF/AHA/SCAI Guidelines on PCI^3^ and 2018 ESC/EACTS guidelines on myocardial revascularization.^4^ On the contrary, benefits of revascularization in stable ischemic heart disease have remained a topic of debate and controversy. Several large clinical trials^5^, ^6^, ^7^, ^8^ have not shown any clear difference in mortality between revascularization and conservative therapy arms. New evidence from long term outcomes of FAME‐2^9^ trial has reported lower incidence of MI with PCI.^9^ With publication of the results of the ISCHEMIA^10^ trial and the 5 years follow‐up results of FAME‐2^9^ trial, we sought to provide in this meta‐analysis a comprehensive and updated assessment of the role of coronary revascularization coupled with medical therapy compared to conservative medical therapy alone (conservative therapy from here‐on) in patients with stable ischemic heart disease.

## 2 METHODS

**FIGURE 1:**
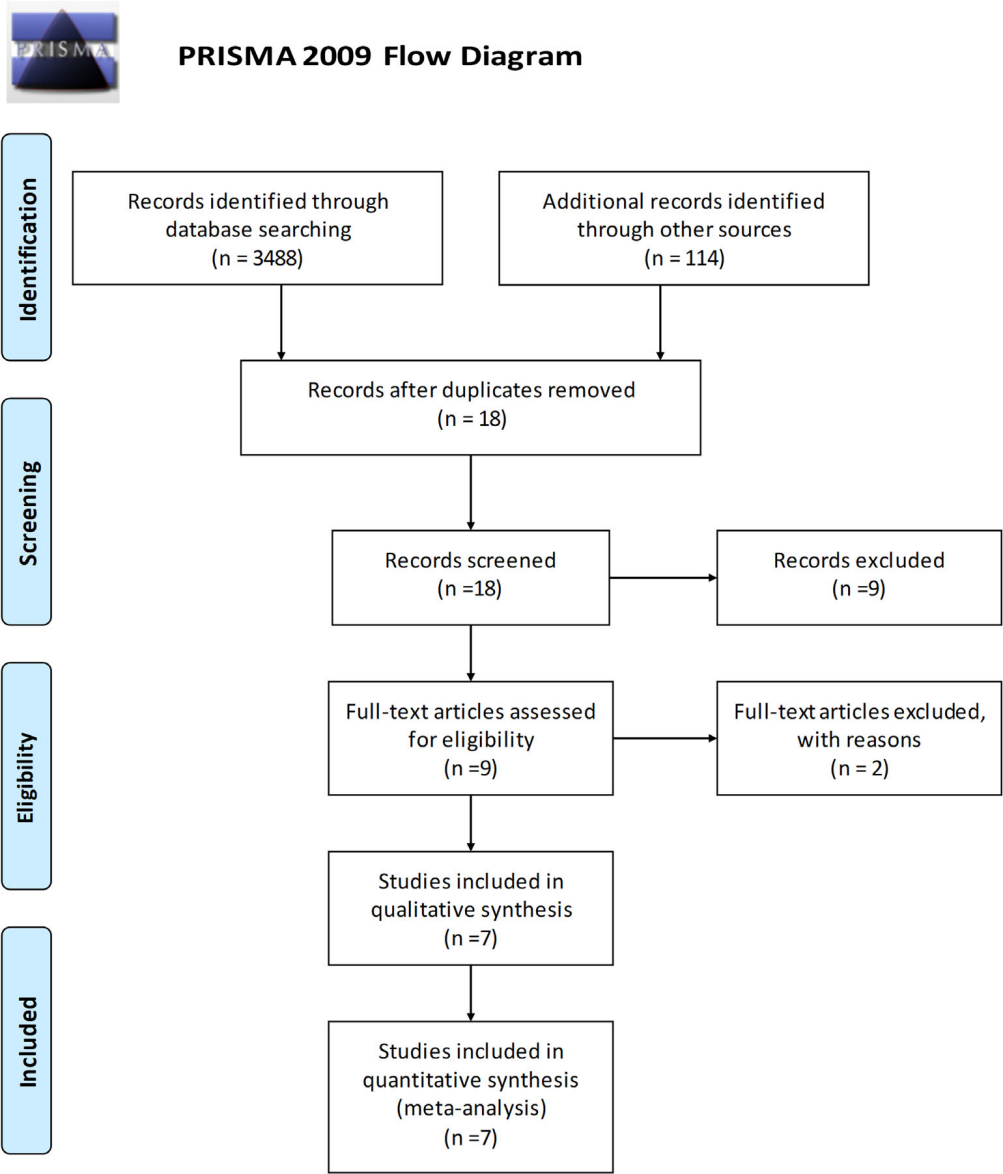
PRISMA flow diagram depicting summary of study selection process

We performed a systematic literature search on PubMed, Cochrane, Embase and Ovid MEDLINE database from January 2000 to June 2020. Search terms included (stable coronary artery disease OR stable angina OR angina) AND (medical therapy OR conservative management OR conservative strategy) AND (PCI OR expanded PCI OR revascularization OR CABG OR surgery). Inclusion criteria were randomized controlled trials (RCTs), trials in humans and English language. Non‐randomized trials, including observational studies, were excluded. Trials investigating treatment strategies other than directly comparing intervention to medical therapy were excluded. This led to exclusion of three major trials namely: ORBITA^11^ trial where the outcomes analyzed were angina relief, rather than hard end points, and follow up period was very short; DEFER trial^12^ which looked at outcomes of safety in deferring intervention in patients where fractional flow reserve (FFR) value was >0.75 (no conservative therapy alone arm) and^3^ the trial by Hambrecht et al^13^ which compared PCI with a 12 month exercise training program (no conservative therapy alone arm). There has been significant evolution in medical therapy for stable CAD. Pivotal trials looking at inhibitors of HMG‐CoA reductase (statins) from 1990's^14^, ^15^ showed a significant improvement in mortality in patients with coronary artery disease. This has led to statins becoming the cornerstone of medical therapy in CAD. On the other hand, trials from 1990 to 2000's which looked at benefit of revascularization to medical therapy; had balloon angioplasty as the primary means of intervention. PCI with stents (preferably drug eluting over bare metal) is now standard practice for revascularization. Based on these significant paradigm shifts in therapies in both arms (revascularization and conservative), we excluded trials before 2000 as they did not reflect the current standard of care. This meta‐analysis was performed using PRISMA statement guidelines,^16^ (Figure 1). Two investigators (A. V., K. K.) independently extracted outcomes into a data collection form. Disagreements were resolved by discussions between the two authors or via reaching a consensus by involving a third investigator (H. C.). The definitions of outcomes were accepted as provided in the studies. In cases of multiple follow up publications in a trial, the longest available follow up was accepted. Ethical/Institutional review board approval was not applicable to our study.

Primary outcome was all‐cause mortality. Secondary outcomes included MACE (death, MI or stroke), cardiovascular mortality, MI and stroke. Additional subgroup analysis for all‐cause mortality was performed comparing^1^ PCI with bare metal stent versus conservative therapy; and^2^ PCI with drug eluting stent versus conservative therapy.

Data was extracted on an intention‐to‐treat basis. Measures of heterogeneity, including Cochran's Q‐statistic and I^2^ index tests were computed. Two‐sided *p* value of ≤ .05 was considered significant. Effect size estimates were presented using the calculated odds ratios and corresponding 95% confidence intervals (CI). Sensitivity analysis was also performed with sequential exclusion of individual studies. We assessed the risk of bias for each study using criteria outlined in the Cochrane Handbook for Systematic Reviews of Interventions^17^ and Forest plot was used for publication bias. All data was transferred into a Review Manager 5 file and statistical analysis was performed using Review Manager (RevMan), Version 5.3, Copenhagen: The Nordic Cochrane Centre, The Cochrane Collaboration, 2014.

## 3 RESULTS

Our literature search yielded seven RCTs. We analyzed a total of 12 013 patients (6109 in revascularization arm and 5904 in conservative medical therapy arm). The mean age was 65 years in both arms. The longest follow up available was 10 years. The baseline characteristics of the included patients in each trial are given in Table 1. Outcomes of interest from included trials are given in (Table S1).

**TABLE 1:** Baseline characteristics of the included trials

Trials (year published)	N (no. of patients)	Age (years)	Male	HTN	DM	Smoking	Ejection fraction (%)	H/o CVA	Type of stent used
TIME (2004)^5^, ^6^: MT INV	148 153	80 80	58% 58%	58% 64%	22% 20%	32% 37%	NA NA	7% 10%	BMS (100%)
MASS II (2010)^8^: MT PCI CABG	203 205 203	60 ± 9 60 ± 9 60 ± 9	69% 67% 72%	55% 61% 63%	36% 23% 29%	33% 27% 32%	68 ± 7 68 ± 8 68 ± 9	N/A	BMS (100%)
COURAGE (2007)^7^: MT PCI	1138 1149	61.8 ± 9.7 61.5 ± 10.1	968 (85%) 979 (85%)	764 (67%) 757 (66%)	399 (35%) 367 (32%)	259 (23%) 260 (23%)	60.9 ± 10.3 60.8 ± 11.2	102(9%) 100 (9%)	BMS (100%)
JSAP (2008)^34^: MT PCI	191 188	64.2 ± 7.6 64.5 ± 7.2	144 (75%) 141 (75%)	121 (63.4%) 119 (63.3%)	76 (39.8%) 76 (40.4%)	23.7% 13.1%	65.8 ± 9.6 64.0 ± 9.7	10 (5.4%) 13 (7.3%)	BMS (100%)
BARI 2 D (2009)^21^: MT PCI and CABG	1192 1176	62.4 62.3	70.4% 70.3%		100% 100%	24.2% 22.9%	57.3% 57.0%	10.0% 9.5%	BMS (56%) and DES (34.7%)
FAME 2 (2018)^9^: MT PCI	441 447	63.9 63.5	338 (76.6%) 356 (79.6%)	343 (77.8%) 347 (77.6%)	348 (78.9%) 330 (73.8%)	90 (20.4%) 89 (18.9%)		28 (6.3%) 33 (7.45)	DES (97%)
ISCHEMIA (2020)^10^: MT INV	2591 2588	64 64	2029 1982	73.4% 73.4%	42.2% 41.4%	N/A	60% 60%	2.6% 3.4%	DES (98%)

### 3.1 Primary pooled analysis

**FIGURE 2:**
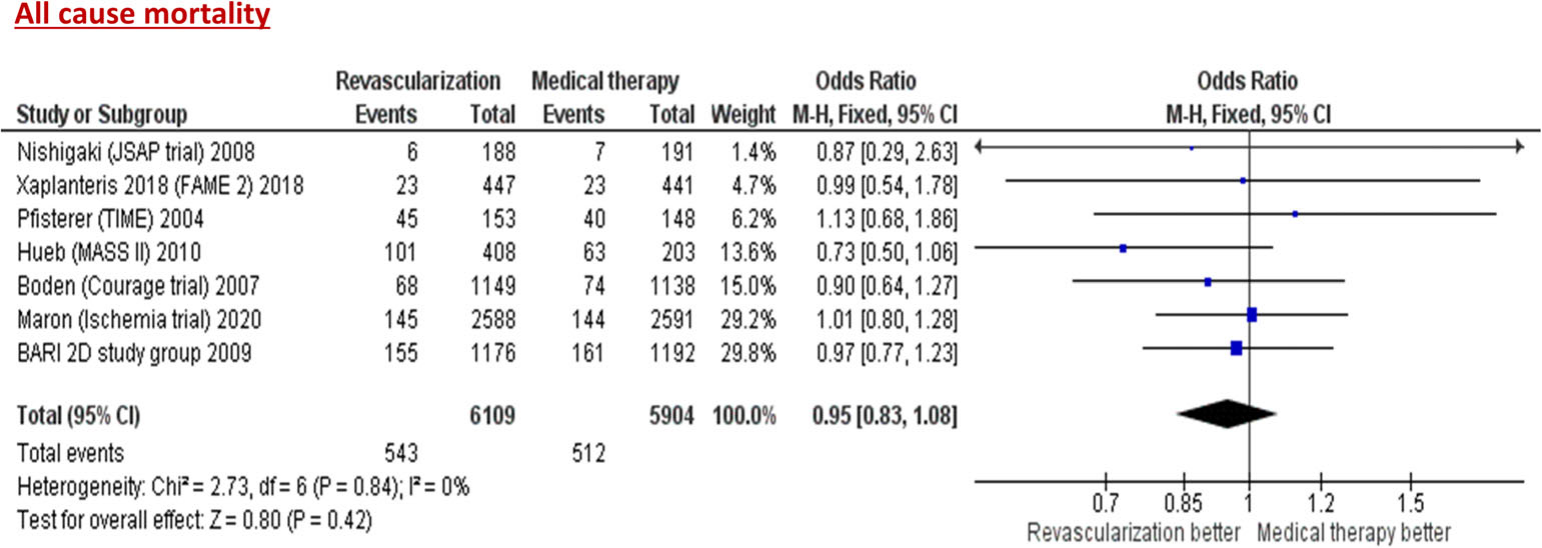
Forest plot of pooled odds ratio (OR) comparing revascularization versus medical therapy for primary outcome of all‐cause mortality. The rectangle represents the point estimate (horizontal line indicates the 95% CI), with its size being proportional to the weight of the study in the meta‐analysis. The diamond represents the pooled estimate (with its size representing the 95% CI)

**FIGURE 3:**
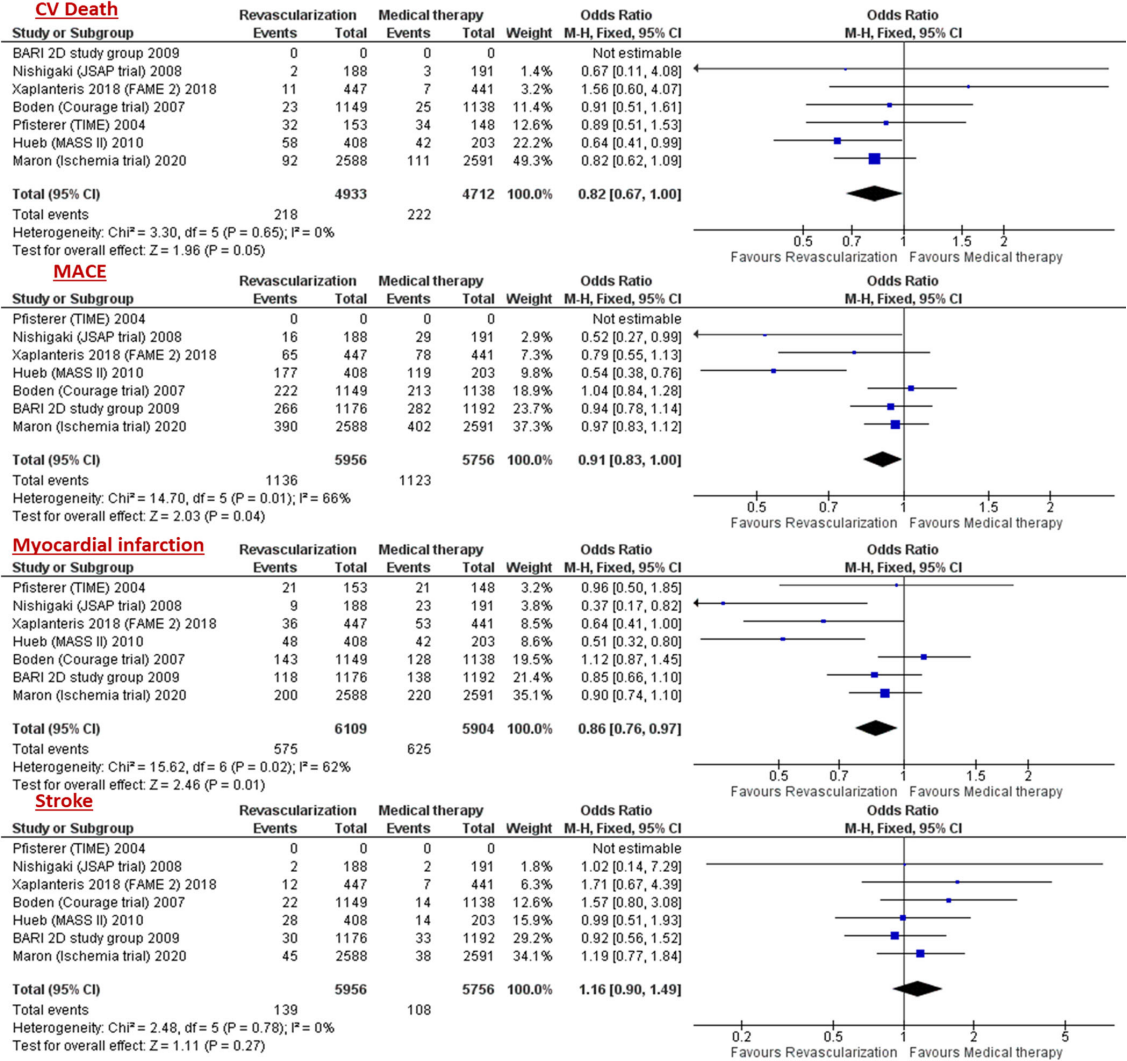
Forest plots of pooled odds ratio (OR) comparing revascularization versus medical therapy for secondary outcomes of cardiovascular death (CV death), MACE, myocardial infarction (MI), and stroke. The rectangle represents the point estimate (horizontal line indicates the 95% CI), with its size being proportional to the weight of the study in the meta‐analysis. The diamond represents the pooled estimate (with its size representing the 95% CI)

The results of this analysis show no statistically significant difference in primary outcome of all‐cause mortality between revascularization and conservative therapy (odds ratio [OR] = 0.95; 95% CI (confidence interval), 0.83–1.08; *p =* .84) (Figure 2). There were statistically significant lower rates of MACE (death, MI, or stroke) (OR = 0.91; 95% CI, 0.83–1.00; *p <* .04), cardiovascular death (OR = 0.82; 95% CI, 0.67–1.00; *p =* .05) and MI (OR = 0.86, 95% CI, 0.76–0.97; *p <* .01) in the revascularization arm when compared to conservative arm. There was no statistically significant difference in stroke (OR = 1.16, 0.90–1.49; *p =* .27) between the two arms. (Figure 3).

### 3.2 Subgroup analysis

**FIGURE 4:**
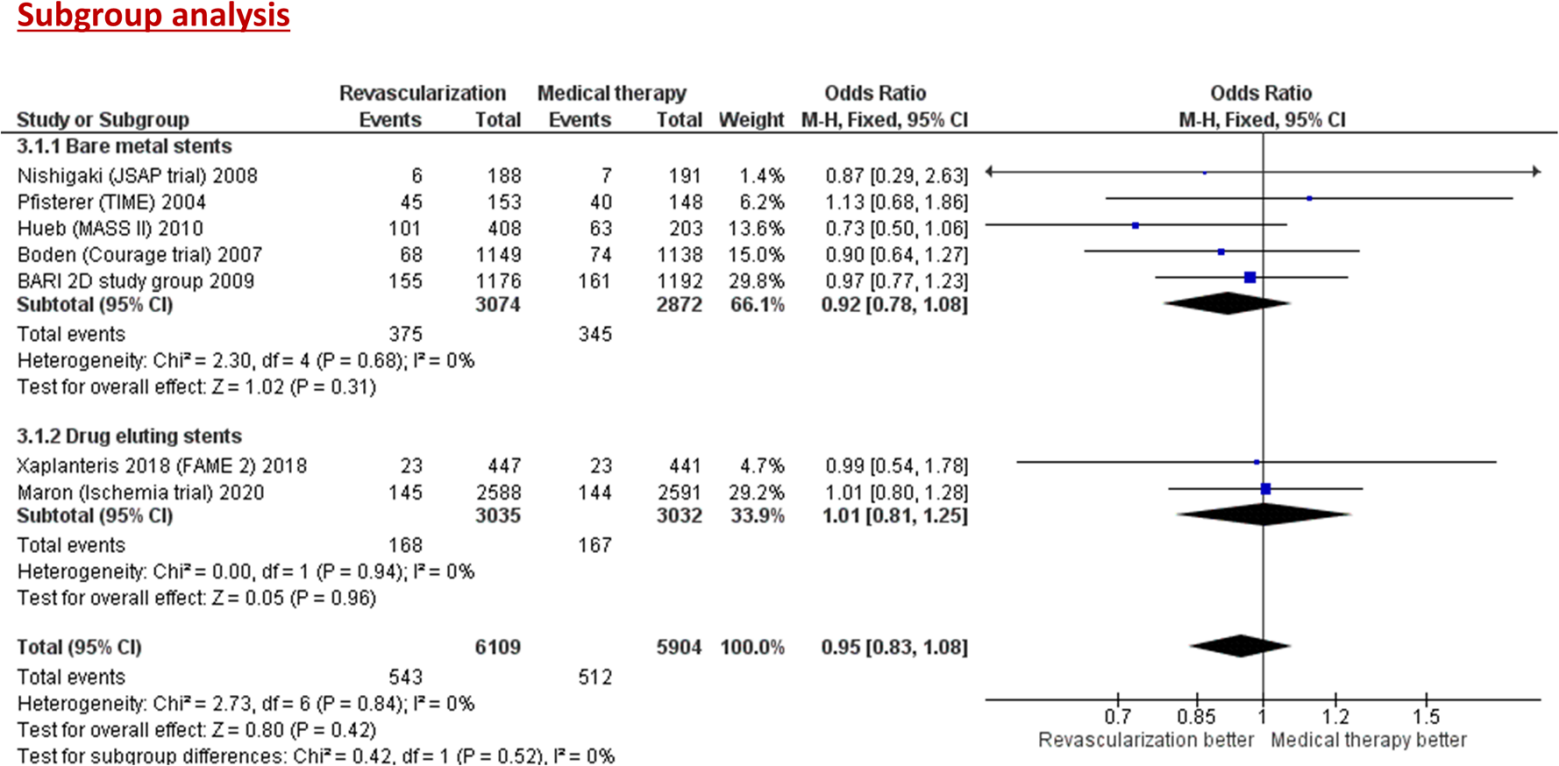
Forest plot of pooled odds ratio (OR) of subgroup analysis comparing revascularization versus medical therapy for primary outcome of all‐cause mortality when stratified based on type of stents (bare metal vs. drug eluting stents). The rectangle represents the point estimate (horizontal line indicates the 95% CI), with its size being proportional to the weight of the study in the meta‐analysis. The diamond represents the pooled estimate (with its size representing the 95% CI)

Subgroup analyses (Figure 4) showed no significant difference in all‐cause mortality when the included trials were stratified based on use of bare metal stents (OR = 0.92; 95% CI, 0.78–1.08; *p =* .31) or drug eluting stents (OR = 1.01; 95% CI, 0.81–1.25; *p =* .52).

## 4 DISCUSSION

This meta‐analysis of over 12 000 patients shows no difference in primary outcome of all‐cause mortality between revascularization with medical therapy and conservative therapy for patients with symptomatic but stable CAD. However, secondary outcomes analyses showed that revascularization is associated with reduced incidence of MACE (death, MI, or stroke) which is driven by a nearly 14% reduction in MI and cardiovascular death when compared to conservative therapy alone.

Current ACC/AHA/AATS/PCNA/SCAI/STS guidelines on stable CAD support coronary revascularization if the patients continue to have ischemic symptoms on optimal medical therapy or in whom revascularization may alter prognosis, such as in patients with reduced EF, where revascularization has a mortality benefit over conservative therapy.^18^ Therefore, current guidelines established PCI or CABG as more of a second line therapy in patients with stable CAD.^19^, ^20^ Although CABG and PCI are acceptable means of coronary revascularization, the trials included in our meta‐analysis have PCI as the predominant means of revascularization, except for BARI‐2D,^21^ MASS‐II^8^ and ISCHEMIA^10^ trial where a significant proportion (32%, 33%, and 26%, respectively) of patients underwent CABG.

Heart disease has remained the leading cause of death in the United States for more than four decades. However, there has been a significant decline in mortality over the years. Aggressive risk factor modification for primary and secondary prevention in CAD has contributed to this remarkable decline in mortality. Previous to the year 2000, trials addressing treatment of stable CAD predominantly used plain old balloon angioplasty (POBA) as the interventional treatment strategy. The outcomes of patients undergoing POBA are marred by the significant rates of restenosis in up to 30%–50% of patients.^22^, ^23^ With the advent of bare metal stents, there was reduced incidence of rate of angiographic restenosis and repeat revascularization when compared to angioplasty alone,^24^, ^25^ although there was no difference in mortality or MI. Drug eluting stents, which were introduced for commercial use in April 2003, have had significant impact on reducing the rates of restenosis and repeat revascularization when compared to bare metal stents.^26^, ^27^ Additionally, intracardiac imaging by utilizing intravascular ultrasound (IVUS) or optical coherence tomography (OCT) in guiding PCI has consistently shown to reduce MACE (cardiac death, target lesion–related MI or ischemia‐driven revascularization).^28^ This benefit was due to a reduction in target lesion revascularization with the use of IVUS, which was sustained to 5 years of follow‐up. Similar results were shown in a meta‐analysis of 31 studies with 17 882 patients where the use of intravascular imaging techniques for PCI guidance reduces the risk of cardiovascular death and adverse events.^29^

Although the field of coronary interventions has come a long way in PCI techniques and equipment, multiple prior meta‐analyses have either shown limited^30^, ^31^, ^32^, ^33^, ^34^ or no difference in outcomes between PCI and conservative therapy.^35^, ^36^ There have been several postulations as to why that is. Despite the improvement in procedural, operator techniques and promising research in stent technology, patients with stable coronary disease have not shown a decrease in death or MI with revascularization. Exclusion of patients with high‐risk anatomical features and enrollment of patients with milder levels of ischemia could have resulted in disproportionate number of patients with low ischemic burden to be enrolled, biasing the results to the null.

The International Study of Comparative Health Effectiveness with Medical and Invasive Approaches (ISCHEMIA)^10^ was specifically designed to overcome the aforementioned limitations of prior data and to determine the effect of revascularization added to medical therapy in patients with stable coronary disease and moderate or severe ischemia. ISCHEMIA trial, failed to show any difference in mortality in either invasive or conservative arm. However, inclusion of patients with less severe CAD than initially proposed remains a major limitation of this trial. One of the major findings in ISCHEMIA was lower incidence of spontaneous MIs on long term follow up in the invasive strategy arm. Cumulatively, however, the increased peri‐procedural MIs in the invasive group negated the effect of late onset MI's and there was no significant difference between both arms at 3 years.

Our analysis updates the current available literature and includes the 5 years follow‐up data from the FAME 2^9^ and lSCHEMIA^10^ trial which compromise more than half of the patients included in the analysis. The emphasis in the analysis was on the longest follow up data available for each trial and the inclusion of trials which are predominantly reflective of contemporary medical practices in both the medical and the invasive arms. A similar meta‐analysis has been published previously by Bangalore et al.^37^ This meta‐analysis included 14 randomized clinical trials with 14 877 patients and had a weighted mean follow up of 4.5 years. They reported no difference in mortality between medical therapy or revascularization. Revascularization was associated with a reduced nonprocedural MI, but also with increased procedural MI. A significant reduction in unstable angina and increase in freedom from angina was also observed with revascularization. Our meta‐analysis differs from the analysis by Bangalore et al predominantly on the basis of inclusion criteria of trials. Older trials such as ACME I,^38^ ACME II,^39^ RITA‐2,^40^ and MASS I^41^ did not have aggressive use of statins for lipid lowering and balloon angioplasty was the predominant means of intervention. We chose to exclude these trials as neither the medical therapy nor the interventions represented current standard of care.

The finding of no survival benefit between the two groups has been consistent among all of the included trials and prior analyses. One of the major findings in our analysis was higher incidence of MI in patients treated with conservative therapy alone. Those results are predominantly driven by ISCHEMIA^10^ and MASS II^8^ data. Our findings also differ from the only recent meta‐analysis that has included the results of the ISCHEMIA trial^36^ which showed no difference in MI rates between either arms in patients with stable CAD. This difference could be related to the fact that the above‐mentioned meta‐analysis included older studies where the use of balloon angioplasty was the predominant practice in the invasive strategy. Our choice to include primary definition of MI's in the ISCHEMIA trial could have also contributed to this difference.

Coronary artery disease should be considered under the category of chronic coronary syndrome to reflect this multi‐faceted disease process that requires interventions on multiple frontiers for a desired response. Atherosclerotic plaque deposition begins in early years of childhood and adolescence as fatty streaks,^24^, ^42^ and takes decades before obstructive lesions impede coronary flow and lead to symptoms. Aggressive medical therapy including high intensity statins has shown favorable results in plaque stabilization and possibly even plaque regression.^43^ This in conjunction with moderate to high intensity exercise training can increase collateral flow index^44^ and help with symptom control. The role of revascularization with PCI or CABG where feasible largely applies to patients with persistent symptoms despite maximal medical therapy.

Our analysis, in alignment with prior individual trials and analyses, failed to show any survival advantage of an initial invasive strategy over conservative medical therapy in patients with stable CAD. The reduced incidence of spontaneous MIs in the revascularization arm is promising. Although numerically, the decrease in spontaneous MI are offset by an increase in peri‐procedural MI, periprocedural MI and spontaneous MI do not carry the same weight in prognostic significance^45^ and should not be considered equivalent as clinical event end points. Large peri‐procedural MI have been shown to be independently associated with increased mortality, however the clinical significance of smaller biomarkers elevations is unclear.^45^, ^46^ Periprocedural MI appears to be more of a marker of baseline patient risk, atherosclerosis burden, and procedural complexity (calcifications, tortuosity).^47^The differences in the definitions and our inability to separate the procedural and spontaneous MIs in many of the analyzed trials can contribute to a decreased apparent clinical benefit of revascularization.

## 5 CONCLUSION

The current meta‐analysis of seven contemporary RCTs did not show any survival advantage of an initial invasive strategy over conservative medical therapy in patients with stable CAD. There was a significantly lower risk of MACE in the revascularization group, predominantly driven by reduced spontaneous M. Shared clinical decision making based on patient's symptoms should be pursued to determine further management options.

## 6 LIMITATIONS

The results of our meta‐analysis need to be interpreted with some inherent limitations. We did not have access to patient level data. Patients with stable ischemic heart disease and low left ventricular ejection fraction or left main disease were excluded from all trials and hence the results should not be applied in those disease states. Although, the aim of our meta‐analysis was to assess the benefit of revascularization for stable CAD, the results predominantly reflect use of PCI, given small number of patients who underwent CABG. We were unable to stratify the results of revascularization based on CABG versus PCI. Choice of CABG over PCI may reflect higher burden of disease and ischemia and expectedly outcomes like stroke and periprocedural MI could be different. Additionally, the type of troponin assay used and definitions of peri‐procedural MI have been varied in different trials and has been mostly linked to biomarker elevation in the absence of addressable symptoms.

## Supporting information


**Table S1**: Outcomes of interest in the included trials.

## Data Availability

The authors declare that all data supporting the findings of this study are available within the article (and its supplementary information files).
