# Supplementary material for: Invasive therapy versus conservative therapy for patients with stable coronary artery disease: An updated meta‐analysis
Source: Clin Cardiol. 2021 Mar 20;44(5):675–82. doi: 10.1002/clc.23592 (PMC8119834; doi:10.1002/clc.23592)
Supplement: Supplementary file 1 — Table S1: Outcomes of interest in the included trials. [file CLC-44-675-s001.docx]

| Trials  (year published) | All-cause mortality | Cardiovascular death | MACE  (death, MI or stroke) | Myocardial infarction | Stroke |
| --- | --- | --- | --- | --- | --- |
| TIME (2004):([5](#_ENREF_5), [6](#_ENREF_6))  MT  INV | 29.3%  33.4% | 34  32 | NA  NA | 14.2%  13.5% | NA  NA |
| MASS II (2010): ([8](#_ENREF_8))  MT  PCI  CABG | 63 (31%)  50 (24%)  51 (25%) | 42 (20.7%)  36 (14.3%)  22 (10.8%) | 119 (58.6%)  88(42.9%)  89(43.8%) | 42 (20.7%)  27 (13.3%)  21 (10.3%) | 14 (6.9%)  11(5.4%)  17(8.4%) |
| COURAGE (2007): ([7](#_ENREF_7))  MT  INV | 74 (6.5)  68 (5.9) | 25 (2.2)  23 (2.0) | 213 (18.7%)  222(19.3%) | 128 (11.1%)  143 (12.4%) | 14 (1.2%)  22 (1.9%) |
| JSAP (2008):  MT  INV | 7 (3.9%)  6 (2.9%) | 3 (1.6%)  2 (1.0%) | 29 (15%)  16 (8.3%) | 23  9 | 2 (1%)  2 (1%) |
| BARI 2 D (2009):([22](#_ENREF_22))  MT  PCI and CABG | 13.5%  13.2% | N/A  N/A | 23.7%  22.6% | 138 (11.6%)  118 (10.0%) | 33 (2.8%)  30 (2.6%) |
| FAME 2 (2018):([10](#_ENREF_10))  MT  INV | 23 (5.2%)  23 (5.1%) | 7 (1.6%)  11 (2.5%) | 78 (17.7%)  65 (14.5%) | 53 (12.0%)  36 (8.1%) | 7 (1.6%)  12 (2.7%) |
| ISCHEMIA (2020):([11](#_ENREF_11))  MT  INV | 144 (8.3%)  145 (9%) | 111  92 | 402  390 | **(Primary definition)**  220  200 | 38 (2.4%)  45(2.3%) |
